# A Narrative Review on the Antitumoral Effects of Selected Mediterranean Plant Products from Southern Italy

**DOI:** 10.3390/ijms262412079

**Published:** 2025-12-16

**Authors:** Adele Elisabetta Leonetti, Loredana Mauro, Francesca De Amicis, Francesca Giordano, Giuseppina Daniela Naimo

**Affiliations:** Department of Pharmacy, Health and Nutritional Sciences, University of Calabria, 87036 Arcavacata di Rende, CS, Italy; adeleelisabetta.leonetti@unical.it (A.E.L.); loredana.mauro@unical.it (L.M.); francesca.deamicis@unical.it (F.D.A.)

**Keywords:** cancer, natural compounds, Mediterranean diet

## Abstract

Natural products are a valuable source of bioactive compounds with established roles in oncology. Their structural diversity and ability to target multiple cancer-related pathways make them promising candidates for anticancer drug development. Increasing preclinical and clinical data highlight their potential not only to exert direct antitumor effects but also to enhance patient tolerance to conventional therapies by reducing side effects and improving treatment adherence. The Mediterranean region, known for its biodiversity and traditional dietary habits, provides a rich array of natural compounds with documented health benefits. Key Mediterranean natural plant products (MNPPs), including bioactives from olive oil, onion, citrus fruits, chili pepper and grapes, exhibit antioxidant, anti-inflammatory, and anti-proliferative properties. This review focuses on the molecular mechanisms of selected MNPPs, such as polyphenols, flavonoids, alkaloids, terpenes, organosulfur and furanocoumarin compounds, which modulate oxidative stress, inflammation, apoptosis, and tumor progression. Evidence from in vitro and in vivo studies supports their role in cancer prevention and as adjuvants in therapy. While further clinical research is needed, these findings suggest that incorporating MNPPs into therapeutic regimens could offer low-toxicity, multi-targeted support in oncology, improving both outcomes and quality of life in cancer patients.

## 1. Introduction

Cancer remains a leading cause of death and disability worldwide, despite major advancements in early detection, treatment, and prevention. Globally, cancer is characterized by uncontrolled cell proliferation, tumor heterogeneity, and invasive malignancies that result in high mortality and social burden. According to the GLOBOCAN 2024 database, an estimated 19.96 million new cancer cases and nearly 9.7 million cancer-related deaths occurred in 2022 [1]. The rise in cancer is associated with population and socioeconomic growth, pollution, aging, unhealthy diet, and lifestyle [2,3]. These underscore the global urgency for more effective and less toxic treatment strategies.

Current standard treatments—such as surgery, chemotherapy, and radiotherapy—have improved outcomes, particularly for early-stage malignancies. However, they remain limited by severe side effects, variable efficacy in advanced disease, and the eventual development of resistance [4]. Adverse effects such as fatigue, myelosuppression, nausea, vomiting, neuropathy, cardiotoxicity, and alopecia often reduce patients’ quality of life and lead to treatment discontinuation [5,6]. Furthermore, the high development costs and low success rates of synthetic chemotherapeutics exacerbate the need for alternative and more sustainable approaches [7].

Recent decades have witnessed the evolution of cancer therapy through combination regimens, targeted therapies, immunotherapy, and personalized medicine based on molecular profiling. These strategies have significantly prolonged survival in certain cancer types. For instance, identifying tumor-specific biomarkers has enabled more tailored treatment decisions and enhanced patient outcomes [8,9]. Nevertheless, chemotherapy remains the cornerstone for many advanced-stage malignancies, despite its systemic toxicity and limitations [10]. Moreover, drug resistance—such as resistance to paclitaxel and cisplatin—is a major clinical challenge that contributes to relapse and poor prognosis [11]. Mechanisms of resistance include genetic mutations, activation of alternative signaling pathways, tumor heterogeneity, and immune evasion [12].

In this context, plant-derived natural products have gained growing attention for their potential to provide effective anticancer agents with lower toxicity and the ability to target multiple molecular pathways [13,14], as well as being considered safer and with low or no toxicity compared to synthetic drugs [15]. Phytochemicals have long served as a foundation in oncology drug development, exemplified by compounds like vinblastine, vincristine (from *Camptotheca acuminata*), paclitaxel (*Taxus brevifolia)*, camptothecin (*Camptotheca acuminata*), and etoposide (*Podophyllum peltatum*), all of which have been successfully integrated into clinical oncology [16,17,18,19].

Beyond the well-established therapeutic agents, recent investigations have increasingly examined the anticancer properties of dietary phytochemicals, many of which come from plants traditionally included in the Mediterranean Diet (MD). This eating pattern has drawn considerable interest from the scientific and medical communities in recent decades. The MD is a nutrient-rich, predominantly plant-based regimen that prioritizes seasonal fruits and vegetables, as well as other plant-derived staples. It also incorporates moderate consumption of fish, poultry, and fermented dairy products, with extra virgin olive oil serving as the main source of beneficial fats. Conversely, the intake of processed red meat and refined sugars is markedly restricted [20].

Extensive research has characterized a wide array of bioactive phytochemicals present in plants typical of the Mediterranean region, many of which exhibit significant anticancer activity. Among the most extensively investigated compounds are curcumin (from *Curcuma longa*), resveratrol (from *Vitis vinifera*), epigallocatechin gallate (EGCG) (from green tea), capsaicin (from chili pepper), oleocanthal, hydroxytyrosol, and oleuropein (from olive oil), bergapten (from *Citrus bergamia*) and onionin A (from onion), all of which modulate key hallmarks of cancer such as oxidative stress, angiogenesis, apoptosis, and metastasis [21,22,23]. Several of these compounds also exhibit chemopreventive properties, modulating DNA repair mechanisms, cell cycle regulation, and inflammatory signaling [24]. Moreover, a number of plant-derived compounds have demonstrated the capacity to reverse or prevent multidrug resistance (MDR), further enhancing their therapeutic value [25,26]. This mechanistic evidence is consistent with the robust epidemiological association between adherence to the MD and reduced cancer incidence.

Interestingly, core plant-based foods integral to the MD, including *Olea europaea* (olive), *Allium sativum* (garlic), *Allium cepa* (onion), *Vitis vinifera* (grapes), *Citrus* (oranges, bergamot, lemon), *Solanum lycopersicum* (tomato), *Capsicum annuum* (pepper), and herbs such as *Origanum vulgare* (oregano) and *Salvia rosmarinus* (rosemary), contain a wealth of polyphenols, flavonoids, terpenoids, carotenoids, and sulfur compounds with demonstrated antioxidant and anticancer properties [27,28,29,30]. While herbal compounds present exciting opportunities for cancer therapy and prevention, challenges remain. These include variability in phytochemical composition, the need for rigorous standardization and clinical validation, and potential herb–drug interactions [31,32]. Moreover, it is important to note that the role of antioxidants in cancer is complex and highly context-dependent. In normal tissues, antioxidants reduce inflammation and DNA damage, thereby exerting chemopreventive effects. However, in established tumors, antioxidants can have dual effects. At low concentrations, they may promote cancer cell survival by maintaining redox homeostasis. At higher concentrations or under specific microenvironmental conditions, some antioxidants paradoxically act as pro-oxidants, increasing ROS to cytotoxic levels and inducing apoptosis [33]. These contrasting effects depend on dose, cell type, and tumor microenvironment, making the therapeutic use of antioxidants in oncology a double-edged sword [34,35]. Further preclinical and translational studies are essential to clarify mechanisms of action, optimize bioavailability, and evaluate safety profiles in well-designed clinical trials [36]. While previous literature has explored the anticancer properties of various Mediterranean natural compounds, including phytochemicals and agri-food by-products, these works often focus on individual diseases, isolated compounds, or specific molecular pathways. In contrast, the present review analyzes the antioxidant and antitumoral potential of Mediterranean food products across multiple tumor contexts, thus providing a broader, more translationally oriented perspective.

## 2. Literature Search Strategy

### 2.1. Study Design

This narrative review is based on a structured but nonsystematic literature search performed in PubMed and Google Scholar. The search focused on studies investigating the anticancer potential of five MNPPs and their major bioactive compounds: oleocanthal, hydroxytyrosol, oleuropein (olive), S-allyl cysteine, onionin A, quercetin (onion), bergapten (bergamot), capsaicin (chili pepper), and resveratrol (grape).

Although this work does not constitute a systematic review and is therefore not intended to follow PRISMA guidelines, elements inspired by the PRISMA, such as explicit reporting of search strategies, eligibility criteria, and study selection, were incorporated to improve transparency, clarity, and reproducibility.

### 2.2. Study Selection

The literature search was conducted in iterative phases from May to September 2025. Keyword combinations were tailored to the known biological activities of each compound. Additional articles were identified through reference lists and citation tracking.

For each plant-based compound, the following generic search strings were used:

- “[compound name]” AND (“anticancer” OR “cancer” OR “tumor” OR “treatment”) to retrieve foundational data on each compound’s ability to affect cancer cell growth and viability.
- “[compound name]” AND (“antioxidant” OR “oxidative stress”) AND (“cancer”) to identify evidence on redox modulation and molecular pathways associated with tumor initiation/progression.
- “[compound name]” AND “in vivo” AND (“cancer” OR “tumor” OR “xenograft” OR “animal model”) to assess available animal models.
- “[compound name]” AND (“preclinical” OR “clinical trial”) AND (“cancer” OR “tumor”) to retrieve translational studies or early-phase human trials, if available.
- “[compound name]” AND (“chemotherapy” OR “chemosensitizer” OR “combination therapy”) AND (“cancer”) to explore interactions with standard anticancer treatments and potential chemosensitizing effects.

### 2.3. Eligibility Criteria

Inclusion Criteria:

- Original research articles
- In vitro, in vivo, or clinical studies
- Published in English
- Relevance to anticancer mechanisms or biological activities of MNPPs.

Exclusion criteria:

- Studies not related to cancer
- Non-original papers (e.g., editorials, commentaries)
- Non-English publications
- Studies lacking mechanistic or biological relevance.

### 2.4. Selection Process

The authors manually screened titles and abstracts. The full texts of potentially eligible papers were evaluated based on their relevance to molecular mechanisms, antioxidant responses, and antitumor activities. As this is a narrative review, no quantitative synthesis or formal PRISMA flow diagram was conducted. However, the selection process adhered to principles of transparency and methodological clarity.

## 3. Natural Plant Products from Mediterranean Diet

The Mediterranean Basin is one of the world’s major biodiversity hotspots. It is home to over 25,000 plant species, around half of which are endemic [37,38]. This exceptional richness of flora stems from a complex biogeographical history, diverse microclimates and long-standing interactions between plants and human activity [39]. Exposure to seasonal drought, intense solar radiation and nutrient-poor soils has favored the evolution of specialized secondary metabolic pathways [40]. Consequently, Mediterranean plants exhibit distinctive phytochemical profiles, rich in phenolic acids, flavonoids, terpenoids, alkaloids, carotenoids, and dietary fiber [41,42]. Many of these compounds exhibit antioxidant, anti-inflammatory, antimicrobial and anticancer properties [42,43].

These bioactive compounds play a key role in the protective effects of the MD, which has been linked to a lower incidence of various cancers, including those affecting the digestive tract, breast, and lungs [44].

Table 1 summarizes key foods from MD, their principal bioactive compounds, and the antitumoral mechanisms documented in major cancer models.

In this narrative review, we focused on five selected plant species (olive, onion, citrus fruits, chili pepper, and grapes) that are not only emblematic of the broader Mediterranean basin but also represent the specific agro-ecological and culinary tradition of Southern Italy.

Furthermore, when compared to the same species growing outside the Mediterranean basin, the Mediterranean populations of the five plant species discussed in this review show a marked increase in phytochemicals, reflecting their adaptations to enhanced photoprotection and oxidative stress management. Table 2 provides an overview of the phytochemical classes and key bioactive molecules that distinguish the selected Mediterranean plant species from non-Mediterranean ones.

### 3.1. Olive Tree Bioactives in Cancer Therapy

Scientific literature reports benefits from olive trees in ancient times, in particular in 1854, when Daniel Hanbury first described that a decoction of olive leaves efficiently reduced malaria-associated fever [70]. Since then, numerous pharmacological studies have highlighted the therapeutic potential of olive-derived extracts, including their ability to alleviate arrhythmias, enhance blood circulation, lower blood pressure, and reduce cancer [71,72].

The extra virgin olive oil (EVOO) from the olive tree is the main fat source in the MD and through mechanical pressing, it provides additional bioactive components, notably oleocanthal, hydroxytyrosol, oleuropein, tyrosol, and monounsaturated fatty acids like oleic acid [73]. These compounds exhibit potent antioxidant activity by scavenging reactive oxygen species (ROS), while also inducing apoptosis in cancer cells and inhibiting inflammation [46]. Large cohort studies such as the EPIC (European Prospective Investigation into Cancer and Nutrition) have shown inverse associations between olive oil intake and the incidence of breast and colorectal cancers [74]. Specifically, EVOO polyphenols can inhibit matrix metalloproteinases (MMPs) and cyclooxygenase-2 (COX-2), which are associated with cancer cell invasion and metastasis. The antitumoral effects of EVOO have been observed in breast, colon, and prostate cancer models [45].

The fruit pulp of the olive is a rich source of polyphenols such as oleuropein and hydroxytyrosol, both of which exhibit potent antioxidant, anti-inflammatory, and anti-proliferative activities (at concentrations ranging from 50 to 100 μmol/L, in HL60 cells, while no effect on apoptosis was observed in freshly isolated human lymphocytes and polymorphonuclear cells). The leaves of the olive tree also contain high levels of oleuropein, along with verbascoside and various flavonoids, contributing to the plant’s therapeutic potential. It has been reported that olive leaf extract reduced cell proliferation of human lymphoblastic leukemia cell line [75]. It was also effective in reducing cell viability of pancreatic and bladder cancer cell lines, blocking cell cycle progression [76,77].

Further in vitro studies have demonstrated that oleuropein inhibits cancer cell proliferation, induces apoptosis, and arrests the cell cycle in multiple cancer cell lines, including breast, colon, and prostate cancers [78,79]. Its antioxidant properties also help reduce oxidative DNA damage, a key factor in carcinogenesis [80]. In breast cancer, oleuropein inhibits estradiol-induced ERK1/2 activation in MCF-7 cells, reducing hormone-driven proliferation [81]. It also induces autophagy and suppresses migration and invasion through the modulation of p62, Beclin-1 and LC3 II/I expression [82]. In both MCF-7 and MDA-MB-231 cells, it increases ROS levels and promotes apoptosis by inhibiting Nuclear Factor kappa-light-chain-enhancer of activated B cells (NF-κB) signaling cascade [83]. In vivo, combined treatment with oleuropein (50 mg/kg), and doxorubicin (2.5 mg/kg) in breast cancer xenografts significantly reduced tumor volume and downregulated NF-κB, Bcl-2 and survivin, enhancing apoptosis more effectively than either compound alone [84]. It has shown efficacy in colorectal, thyroid and lung cancer models [78,85,86,87].

Despite oleuropein being determined in plasma, it undergoes extensive metabolism to hydroxytyrosol and other products [88].

Oleocanthal, a phenolic aldehyde unique to EVOO, has anticancer activity in various tumor cell lines, especially breast cancer, melanoma, and multiple myeloma cells. In breast cancer (MCF-7, MDA-MB-231 and BT-474) inhibits proliferation, migration and G1/S cell cycle progression through suppression of Hepatocyte Growth Factor (HGF)-induced c-Met activation, without affecting non-tumorous cells (MCF10). The IC_50_ values for (-)-oleocanthal treatment in HGF-supplemented media were 10.9, 20.1 and 25.4 µM in MDA-MB-231, MCF-7 and BT-474 breast cancer cells, respectively. However, the IC_50_ values for (-)-oleocanthal treatment in HGF-free media were 16.2, 40.8 and 58.4 µM in MDA-MB-231, MCF-7 and BT-474 breast cancer cells, respectively. These results indicate that (-)-oleocanthal treatment inhibits HGF-dependent MDA-MB-231, MCF-7 and BT-474 cell growth in a dose- and time-responsive manner compared to cells in the vehicle-treated control groups [89]. In melanoma cells (501Mel and A375), it reduces viability in a dose-dependent manner through COX inhibition and downregulation of Bcl-2, ERK1/2 and AKT signaling [90,91]. The downregulation of the latter pathway is implicated in BRAF inhibitor resistance in melanoma, suggesting a potential chemosensitizing role for oleocanthal in resistant tumors [92]. Oleocanthal (0.2, 2 and 20 μM) works as a COX-1 and COX-2 inhibitor, mirroring the anti-inflammatory effect of ibuprofen, and has been shown to selectively induce apoptosis in human cancer cells by disrupting lysosomal membranes [93]. Moreover, oleocanthal (60 µM) potentiates chemosensitivity in gastric cancer cells resistant to cisplatin, 5-fluorouracil, and paclitaxel in vitro [94]. Combined with lapatinib, a Human Epidermal Growth Factor Receptor 2 (HER2)/EFR inhibitor, it reduces proliferation of HER2-positive breast cancer cells (BT-474, SKBR-3), reducing lapatinib dose (using 12 µM oleocanthal with 30 nM Lapatinib in BT-474 cells and 15 µM with 60 nM in SKBR-3) [95]. In breast cancer cells with HER2 overexpression, it synergized with trastuzumab, enhancing cancer cell death and targeting HER2 and FAS signaling pathways. Its ability to potentiate chemotherapy is also reported in other cell lines, like gastric cancer cells, liver, and colon [94,96]. In vivo studies using xenograft mouse models have confirmed the tumor-suppressive effects of both oleuropein and oleocanthal, leading to reduced tumor volume and angiogenesis [21]. However, due to the limited pharmacokinetic data in humans, oleocanthal is difficult to detect in plasma following administration, suggesting that its biological effects may instead be mediated by its metabolites [97].

Hydroxytyrosol, a phenolic compound, is rapidly absorbed and over 90% is excreted with the urine; its absorption strongly depends on the food matrix, with oily matrices like EVOO enhancing bioavailability compared to aqueous vehicles [98,99]. It has chemotherapeutic effects through modulation of several oncogenic pathways, including growth factor receptors (Epidermal Growth Factor Receptor, EGFR; Insulin-like Growth Factor 1 Receptor, IGF-1R) [85,100,101], receptor-associated proteins [102,103], and interleukin signaling [104]. It inhibits cyclin D1, leading to G1/S cell cycle arrest, particularly in MCF-7, MDA-MB-21 breast cancer cells, CaCo2 colon cancer cells and TPC-1, FB-2, and WRO thyroid cancer cell lines at a concentration of 324 µM [103,105,106]. It can reduce ROS generation, leading to apoptotic cell death and mitochondrial dysfunction, as shown in colon (DLD1) and prostate (PC3) cancer models [107,108]. Hydroxytyrosol selectively inhibits prostate cancer cell proliferation (LNCaP and C4-2) through downregulation of Cyclins D1/E and CDKs 2/4 and upregulation of p21^WAF1^/p27^KIP1^, leading to G1 arrest. Apoptosis is promoted via caspase activation, Poly (ADP-ribose) Polymerase (PARP) cleavage and increased BAX/Bcl-2 ratio (at increasing concentrations of 100, 200 and 300 µM) [109]. It also inhibits AKT/Signal Transducer and Activator of Transcription 3 (STAT3)/NF-κB signaling in androgen-sensitive prostate cancers [110,111]. In colorectal adenocarcinoma cells (HT-29, WiDr, CaCo_2_), it significantly downregulated EGFR expression via ubiquitin-mediated proteasomal and lysosomal degradation [101]. Additionally, hydroxytyrosol has entered early-phase clinical trials for its antioxidant and cardioprotective effects, with emerging interest in its potential adjunct role in cancer prevention and therapy [112].

### 3.2. Antitumor Effects of Onion-Derived Bioactive Compounds

The Mediterranean diet is rich in vegetables, and among the most commonly used is the onion. In particular, the Tropea onion stands out as one of the most renowned varieties, both for its flavor and its health properties. The outer layers and red onion varieties are rich in flavonoids, in particular quercetin, organosulfur compounds, such as S-allyl cysteine and onionin A, and other phenolic constituents. These bioactive molecules exert anti-proliferative, pro-apoptotic, antioxidant, and anti-inflammatory effects, making them promising adjuvants in cancer therapy.

In vitro, quercetin-rich extracts and quercetin glucosides inhibit proliferation in diverse cancer cell lines, including breast (MCF-7), colon (HT-29), prostate (PC-3), liver (HepG2), and adrenocortical carcinoma (H295R, SW-13). After ingestion, it is rapidly absorbed and extensively metabolized into conjugated forms such as quercetin-3′-sulfate and quercetin-3′-glucuronide, which appear in plasma within 1–2 h and are excreted within 4–8 h. Its absorption is enhanced when consumed as onion peel extract and in the presence of dietary fats. Conjugated metabolites, rather than the aglycone, predominate systemically; therefore, aglycone-centric mechanisms observed in vitro do not directly mirror plasma exposure. However, several processes allow partial translation in vivo. Quercetin conjugates have the capacity to undergo site-specific deconjugation in β-glucuronidase-rich tissues, such as those involved in detoxification and repair (liver, kidney, intestinal epithelium, spleen, lymph nodes, thymus, ovary, and preputial gland), thereby regenerating the aglycone locally at biologically relevant levels. It has been demonstrated that certain conjugates exhibit intrinsic bioactivity, encompassing effects on redox balance, endothelial function, and cell signaling. Conjugated species may enter cells via dedicated transporters and be deconjugated intracellularly, yielding transient intracellular aglycone pools. Collectively, these mechanisms support the possibility that aglycone-based anti-proliferative effects can still occur in vivo, but through metabolite-dependent and tissue-specific activation, rather than direct systemic exposure [113].

Mechanistic studies in vitro show quercetin induces G1 or G2/M cell cycle arrest; activates apoptosis by modulating BAX/Bcl-2 ratio, activating caspases and p53, and causing mitochondrial membrane depolarization; and reduces ROS in treated cells. It also inhibits the Phosphatidyl Inositol 3-Kinase (PI3K)/AKT and MAPK/Extracellular signal-Regulated Kinase (ERK) signaling pathways involved in tumor progression and chemoresistance [114]. Moreover, in multidrug-resistant human breast cancer cells (MCF-7/ADR), quercetin (10 μM) sensitizes cancer cells to chemotherapeutics like cisplatin and doxorubicin by interfering with drug-resistance pathways, such as downregulation of P-glycoprotein. In vivo, quercetin suppresses tumor growth and angiogenesis in murine models; it reduces oxidative DNA damage and enhances the activity of detoxification enzymes (e.g., glutathione S transferase), contributing to its chemopreventive potential [115]. Quercetin (30 μmol/L) enhances the therapeutic efficacy of temozolomide in glioblastoma models (U87 and U251) by promoting apoptosis and autophagy [116].

Onionin A is a sulfur-containing compound uniquely isolated from onion bulbs that lacks pharmacokinetic characterization in both humans and animals, limiting current understanding of its systemic availability and therapeutic potential. In vitro, it inhibits proliferation and induces G2/M cell cycle arrest in ovarian, liver and pancreatic cancer cell lines [114] and suppresses STAT3 activation, which is commonly involved in tumor growth and immune evasion. Moreover, it reduces the population of myeloid-derived suppressor cells (MDSCs), thus reversing tumor-mediated immune suppression [117]. In murine models of ovarian cancer, onionin A reduced tumor burden and prolonged survival time and inhibited tumor angiogenesis and macrophage infiltration [118]. It has been reported that onionin A potentiates the antitumor effects of paclitaxel in ovarian cancer cells by enhancing tumor cell sensitivity and modulating the tumor microenvironment [117].

S-allyl cysteine and other organosulfur compounds, instead, act as ROS scavengers, reducing oxidative stress in normal cells and protecting healthy tissue by neutralizing ROS [119]. In contrast, they promote ROS-mediated apoptosis in cancer cells, leading to DNA damage, mitochondrial dysfunction, and activation of apoptotic pathways through Jun-N-Kinase (JNK) signaling [120]. These compounds modulate cell cycle regulatory proteins by downregulating cyclin D1 and upregulating the CDK inhibitor p21^WAF1^, resulting in cell cycle arrest in cancer cells, like colon, bladder, and lung (effects were observed at a concentration of 300 μmol/L) [118,120,121]. S-allyl cysteine interferes with carcinogen activation via cytochrome P450 enzyme inhibition in animal models [122].

### 3.3. Anticancer Potential of Bergamot-Derived Bergapten

*Citrus bergamia*, commonly known as bergamot, is a small citrus tree believed to be a hybrid between *Citrus aurantium* (bitter orange) and *Citrus limon* (lemon). It is predominantly cultivated in the coastal areas of Calabria, in Southern Italy, where the unique climatic conditions favor the production of high-quality fruits rich in aromatic compounds. In addition to its widespread use in the perfume and food industries through its essential oil, bergamot holds a notable place in the Mediterranean diet, where it is consumed in the form of juices, extracts, and traditional preparations [123]. This dietary inclusion contributes to the overall antioxidant and anti-inflammatory properties associated with Mediterranean nutritional patterns.

Bergamot contains a variety of biologically active secondary metabolites, including flavonoids and furanocoumarins, among which bergapten (5-methoxypsoralen, 5-MOP) is one of the most studied [124,125]. Belonging to the linear furanocoumarin subclass, bergapten is characterized by a coumarin core fused with a furan ring and a methoxy substituent, conferring distinctive photoreactive and lipophilic properties.

Pharmacokinetic studies have reported good absorption of bergapten after oral administration, with absolute bioavailability ranging from approximately 70% to 94% in animal models. Interestingly, in vitro analysis performed on Caco-2 cells highlighted a moderate intestinal permeability, while in vivo studies reported rapid absorption and wide tissue distribution, particularly in the liver, heart, brain, testis, uterus, and skin. Notably, it has been observed that bergapten is also able to cross the blood–brain barrier, suggesting potential central nervous system activity. After oral administration, bergapten is mainly metabolized in the liver and excreted via feces, with the production of multiple metabolites through oxidation and conjugation pathways. Importantly, it has been shown that bergapten inhibited several CYP450 enzymes (e.g., CYP3A4, CYP1A1, CYP1A2), influencing drug–drug interactions and oral bioavailability of co-administered compounds [123].

It has been extensively investigated for its anti-inflammatory, antioxidant, and cytoprotective activities in various experimental models involving oxidative stress, immune responses, and tissue injury.

More recently, the potential of bergapten in oncological research has attracted growing attention, particularly due to its ability to modulate key cellular processes such as apoptosis, pyroptosis, ferroptosis, and mitochondrial function at concentrations of 5, 10, and 20 μM [126]. These pleiotropic effects, combined with its well-documented photosensitizing capacity, make bergapten a promising candidate for cancer prevention and therapy, especially within the context of photodynamic and photochemotherapeutic approaches [127,128,129]. Supporting this potential, studies have shown that ultraviolet-activated bergapten can significantly disrupt survival signaling in breast cancer cells, enhancing cell death through the modulation of critical regulatory pathways. This highlights the compound’s ability to trigger DNA damage and apoptosis in malignant cells, further reinforcing its therapeutic relevance in light-based anticancer strategies. Several other studies and reviews have further emphasized the potential of bergapten and related furocoumarins as natural photosensitizers, highlighting their ability to generate ROS, intercalate with DNA upon UV exposure, and trigger cell death mechanisms in various cancer models. The relevance of this compound in both photodynamic applications and diet-related cancer risk modulation has also been increasingly recognized in recent pharmacological and oncological literature [130,131,132].

Taken together, these findings delineate the well-established photoactivated anticancer actions of bergapten, which depend on UV-induced DNA intercalation, ROS generation, and apoptotic signaling. However, because these mechanisms depend on light exposure, any potential clinical application would require careful control of UV dose, treatment timing, and phototoxicity risk. The latter remains a known concern for linear furocoumarins. Sunlight exposure can significantly impact both therapeutic and adverse effects and must therefore be considered in translational settings [130,133].

Unlike the UV-dependent pathways described above, a growing body of evidence shows that bergapten has non-photoactivated and UV-independent anticancer effects. These activities occur in the absence of light stimulation, bypassing the phototoxicity concerns associated with its photosensitizing properties.

In human breast cancer cells, Panno et al. provided early evidence that bergapten can induce apoptosis without the need for UV activation. The compound was shown to enhance the gene expression of p53, a key tumor suppressor involved in DNA repair and cell cycle arrest, thereby promoting programmed cell death [134]. This photo-independent effect broadens the potential therapeutic use of bergapten beyond phototherapy settings. Building on this, subsequent studies have deepened this perspective. Bergapten has been shown to activate autophagy in breast cancer cells by upregulating Phosphatase and TENsin homolog (PTEN) expression, contributing to the suppression of oncogenic PI3K/AKT signaling [135]. In parallel, another investigation demonstrated that bergapten (5, 10, 20 and 50 μM) triggers metabolic reprogramming in breast cancer cells, shifting them away from a glycolytic phenotype and potentially sensitizing them to further therapeutic interventions [136]. Moreover, bergapten was also found to trigger estrogen receptor (ER) depletion through SMAD4-mediated ubiquitination, further undermining key survival mechanisms in hormone-responsive breast tumor cells [137].

Beyond breast cancer, the anticancer activity of non-UV-activated bergapten extends to multiple tumor cell lines. In colorectal cancer cells, bergapten (30 and 50 μM) induces G1 cell cycle arrest and a pro-apoptotic cascade involving the p53/p21^WAF1^/PTEN axis, highlighting its ability to modulate crucial checkpoints of cell proliferation and survival [138]. A 2023 study investigating the non-UV-activated form of bergapten in several tumor cell lines, including osteosarcoma (Saos 2/HOS), colorectal carcinoma (HT-29/SW 620), and multiple myeloma (RPMI 8226/U266), reported dose-dependent cytotoxicity, with Saos 2 cells being particularly sensitive (IC_50_ ~40 μM). The mechanism involved G2 phase cell cycle arrest, mitochondrial membrane depolarization, and activation of caspase 9 and 3, along with an increased BAX/Bcl-2 ratio and decreased AKT phosphorylation. Notably, bergapten showed limited toxicity toward normal fibroblasts and osteoblasts, indicating selectivity for cancer cells. The HT-29 (colorectal adenocarcinoma cells) (IC_50_ = 332.4 μM), SW 620 (metastatic colorectal carcinoma) (IC_50_ = 354.5 μM), and HOS (osteosarcoma cell line) (IC_50_ = 257.5 μM) cells were characterized by moderate sensitivity, while RPMI8226 (multiple myeloma) (IC_50_ = 1272 μM) and U266 (multiple myeloma) (IC_50_ = 1190 μM) were particularly drug-resistant [139]. In non-small cell lung cancer (NSCLC), bergapten has been shown to induce G1 arrest associated with p53-mediated signaling, and network pharmacology combined with molecular docking further elucidated its interaction with multiple targets involved in NSCLC progression [140]. Interestingly, in hepatocellular carcinoma, bergapten was reported to inhibit liver carcinogenesis through modulation of LXR/PI3K/AKT and IDOL/LDLR pathways both in vitro and in vivo, suggesting a role in tumor metabolism and cholesterol regulation [141]. Additionally, in pancreatic cancer models, novel bergapten derivatives were designed, synthesized, and biologically evaluated, revealing potent antitumor activity and supporting structural optimization for therapeutic use [142]. In a separate 2025 study, bergapten (10 μM/mL and 15 μM/mL) was shown to inhibit proliferation of human papillary thyroid cancer cells via modulation of the GSK-3β, PI3K, and AKT pathways, along with apoptosis induction [143].

From a clinical perspective, this dual behavior highlights an important distinction. While photoactivated bergapten shows promise for controlled photodynamic applications, its intrinsic photoreactivity poses a risk of phototoxicity, especially when exposed to natural sunlight. Conversely, non-photoactivated mechanisms may offer therapeutic opportunities with a potentially safer profile, provided light exposure is managed properly. Understanding this dichotomy is crucial for future translational and clinical research.

These findings suggest a multi-pathway, multi-tumor effect for bergapten, reinforcing its potential as a natural anticancer agent.

In conclusion, bergapten emerges as a multifunctional, diet-derived anticancer agent capable of affecting diverse tumor cell lines through mechanisms such as p53/PTEN axis activation, autophagy induction, ER degradation, metabolic interference, and signaling pathway modulation, with or without UV activation. These properties support its promising role in cancer prevention and therapy, particularly within the context of the Mediterranean diet.

### 3.4. Role of Capsaicin in Cancer Therapy

The chili pepper is rich in capsaicin, which is the main pungent alkaloid. It is a member of a family of compounds that includes dihydrocapsaicin, homocapsaicin, homodihydrocapsaicin, nordihydrocapsaicin, capsaicin esters, dihydrocapsaicin ester, nordihydrocapsaicin esters, capsanthin-β-D-glucoside and dihydrocapsanthin-β-D-glucoside [144]. Capsaicin has been demonstrated to exert analgesic, antioxidant, cardioprotective, anticancer and thermogenic effects, and it can promote weight loss [145]. The majority of these effects are mediated by the TRPV1 receptor (Transient Receptor Potential Cation Channel Subfamily V member 1), to which capsaicin binds specifically [146]. A number of studies have reported that the half-life of capsaicin is relatively short in various organs, including liver, kidney, intestine, lung and blood [147]. However, the administration of capsaicin is associated with different side effects, including gastrointestinal discomfort, such as stomach pain, irritation, gastrointestinal cramps, nausea, and vomiting. Nevertheless, two strategies have been investigated to overcome these effects. Firstly, the encapsulation of capsaicin in drug delivery systems and, secondly, the use of non-pungent capsaicin analogs, which preserve the antitumor activities [148]. Al-Samydai et al., to enhance capsaicin’s pharmacokinetic properties, loaded the molecules into nanoliposomes (IC_50_ of 21.52 ± 10.90 μmole) model and demonstrated that this system enhanced stability, selectivity and safety compared to the capsaicin alone (IC_50_ of 515.55 ± 207.8 μmole), as well as higher anticancer activity, inducing apoptosis in a dose-dependent manner in various cancer cell lines (MCF7, MDA-MB 231, K562, PANC1 and A375) [149].

In recent years, capsaicin has garnered growing scientific attention for its anticancer and chemopreventive potential [150]. Extensive research has demonstrated that capsaicin can disrupt several key pathways involved in cancer development and progression. These include the induction of apoptosis and cell cycle arrest [52], as well as the activation of autophagic processes [151]. Mechanistically, capsaicin has been shown to promote the generation of reactive oxygen species (ROS) and modulate critical signaling cascades, such as NF-κB, STAT3, MAPK, PI3K-AKT, Hedgehog, and β-catenin, while also enhancing the activation of ASK1 [152].

In lung cancer cell lines, capsaicin exerts anti-proliferative and pro-apoptotic effects. It inhibits proliferation, induces apoptosis and autophagy, and suppresses angiogenesis. Notably, it (50 µM) activates the E2F4 pathway in small cell lung cancer (NSCLC) [153], promotes mutant p53 degradation via autophagy (IC_50_ of 200 μM) [154], and triggers apoptosis through the TRPV6-calcium-calpain axis [155]. Moreover, capsaicin (at concentrations of 1 and 10 μM) induces G0/G1 cell cycle arrest and apoptosis by suppressing HER2, EGFR, ERK, and cyclin D1 while increasing p27^KIP1^ and caspase activity [156]. Moreover, it promotes apoptosis via both caspase-dependent and -independent pathways [157] and activates autophagy through p38 MAPK signaling in malignant and normal breast epithelial cells [158]. Additionally, capsaicin (200 μM) triggers mitochondrial-mediated apoptosis and PARP cleavage in MCF-7 and BT-20 cells [159].

In colorectal cancer cells, the role of capsaicin remains controversial. Some studies report tumor-promoting effects via ROS generation, AKT/mTOR and STAT3 pathway activation, and tNOX upregulation (at 100 µM for SW480 and CT-26 cell lines and at 12.5 µM for HCT116 cell line) [160], while others demonstrate its antitumor action via PPARγ activation [161], ROS-mediated mitochondrial disruption [162], nitric oxide induction (100 μM of capsaicin and/or 50 μM of resveratrol) [163], and p53 stabilization [164]. It also possesses immunomodulatory activity, affecting cytokine production in co-cultures of colon cancer cells and PBMCs. 200 µM of capsaicin suppresses TNF-α, IL-1β, IFN-γ, IL-10, and IL-1ra while stimulating IL-6 at lower concentrations, suggesting dose-dependent immune modulation [165].

In stomach cancer, capsaicin exhibits concentration-dependent effects: low intake may offer protection, while high intake has been associated with increased cancer risk, particularly in genetically predisposed individuals infected with *Helicobacter pylori* [166]. Mechanistically, it promotes apoptosis and proliferation arrest via cytochrome c release, p53 activation, and MAPK signaling in human gastric cancer cells [167] and inhibits migration by downregulating tNOX and POU3F2 in human gastric carcinoma cells [168]. It also attenuates *Helicobacter pylori*-induced inflammation, suggesting a potential role in the chemoprevention of gastritis-associated gastric cancer. Capsaicin exerts anticancer effects through both TRPV1-dependent and TRPV1-independent mechanisms. In the TRPV1-dependent pathway, capsaicin activates the TRPV1 channel, inducing Ca^2+^ influx, ROS generation, ER stress, autophagy, and JNK activation, leading to apoptosis [169,170,171]. Conversely, low-dose TRPV1 activation may stimulate PI3K/AKT signaling and promote proliferation in androgen-sensitive prostate cancer cells [150]. In TRPV1-independent mechanisms, capsaicin directly disrupts mitochondrial function, increases ceramide accumulation, and inhibits AKT, NF-κB, and ERCC1 signaling, enhancing sensitivity to chemo- and radiotherapy [172,173,174].

In prostate cancer cells, capsaicin exerts anti-proliferative and pro-apoptotic effects through both TRPV1-dependent and -independent pathways. These effects involve ROS production, endoplasmic reticulum stress, ceramide accumulation, autophagy induction, and JNK activation [169,170,171]. Its action is dose- and androgen-sensitivity dependent: low doses may stimulate proliferation in androgen-sensitive cells via PI3K-TRPV1 signaling, while higher concentrations inhibit growth independently of TRPV1 [175].

One of the most promising aspects of capsaicin’s pharmacological profile is its ability to enhance the efficacy of conventional chemotherapeutic agents. A growing body of evidence supports the notion that capsaicin can sensitize cancer cells to various anticancer drugs, leading to synergistic anticancer effects both in vitro and in vivo.

Capsaicin has demonstrated synergistic effects with various chemotherapeutic agents across multiple tumor cell lines. In cholangiocarcinoma and gastric cancer, it enhanced the cytotoxicity of 5-fluorouracil (5-FU) by inhibiting autophagy via the PI3K/AKT/mTOR pathway and reducing 5-FU’s IC_50_. The IC_50_ of QBC939 cells for 5-FU in combination with capsaicin (20, 40, 80 μM) was significantly decreased from 126 μM to 35 μM, and significant synergy with capsaicin was found at 40 μM [176]. In SCLC, this compound potentiated camptothecin-induced apoptosis through intracellular Ca^2+^ elevation and calpain activation [177]. Co-treatment with cisplatin (DDP) in osteosarcoma and gastric cancer cell lines increased apoptosis and suppressed NF-κB signaling [178,179], while capsaicin also sensitized bladder cancer cells to pirarubicin by inducing cell cycle arrest via TRPV1 activation [180]. In B-cell lymphoma, this compound restored etoposide sensitivity by enhancing CD40-mediated apoptosis [181]. While capsaicin generally acts as a chemosensitizer, one study noted that it promoted resistance in bladder cancer to mitomycin C, gemcitabine, and doxorubicin via Hedgehog-dependent EMT and autophagy (IC_50_ of 300 μM) [182]. However, combinations of capsaicin with other dietary compounds improved gemcitabine efficacy in preclinical in vivo model of pancreatic cancer [183]. In hepatocellular carcinoma cell lines, capsaicin synergizes with sorafenib to inhibit proliferation and induce apoptosis through TRPV1-independent mechanisms by potentiating ERK signaling [174]. Capsaicin (25, 50 and 100 μM) also boosted the anticancer activity of erlotinib in NSCLC by suppressing ERCC1 and AKT signaling [172]. Finally, capsaicin (1–10 µM) sensitized prostate cancer cells to radiation therapy (RT) by inhibiting NF-κB, with no added toxicity in normal tissues. Clinical observation supports its role in prolonging PSA [173].

Collectively, these findings underscore the potential of capsaicin as a valuable adjuvant in cancer therapy. Its natural origin and ability to augment the effectiveness of standard treatments suggest that dietary phytochemicals could play a supportive role in oncology. Nevertheless, the precise molecular mechanisms driving capsaicin-induced drug sensitization remain to be fully elucidated and warrant further investigation.

### 3.5. Resveratrol in Cancer Therapy

Wine is traditionally regarded as one of the components of the Mediterranean diet. Several studies have highlighted how the moderate consumption of wine, particularly red wine during meals, can exert beneficial effects on human health, especially on the cardiovascular system. This is due in part to the presence of bioactive compounds, named polyphenols, which are abundant in red wine and are known for their antioxidant, anti-inflammatory, and cardioprotective properties [184].

Polyphenols in wine are broadly classified into two main groups: flavonoids (including anthocyanins, flavanols, and flavonols) and non-flavonoids. The latter category includes phenolic acids, lignans, and stilbenes, among which the most studied and biologically relevant is resveratrol [185]. Particularly, resveratrol is predominantly found in the skin of red grapes (Vitis vinifera), where it acts as phytoalexin, a compound produced by grapevines in response to environmental stress and pathogenic invasion, which significantly increases its amount [186]. Although resveratrol concentration in wine is relatively low, usually only a few milligrams per liter, the repeated intake through moderate, regular wine consumption could contribute to its observed biological effects.

Chemically, resveratrol is known as 3,4′,5-trihydroxy-trans-stilbene, consisting of two aromatic rings connected by a double bond and bearing three hydroxyl groups that are crucial to its biological activity [187]. The double bond enables isomerization under UV irradiation, with the trans-isomer being not only more abundant than the cis-isomer but also more biologically active. This structural configuration underlies its antioxidant, anti-inflammatory, cardioprotective, neuroprotective, and anticancer effects [187]. In the context of cancer prevention and therapy, resveratrol has emerged as one of the principal bioactive compounds contributing to the potential health benefits of wine.

Extensive in vitro research has highlighted the important role of resveratrol in the early stages of carcinogenesis, largely due to its strong antioxidant activity. Interestingly, a dual antioxidant effect of resveratrol has been reported, depending on cellular context. In cancer cells, it has been shown to prevent oxidative stress-induced DNA damage by disrupting redox homeostasis, leading to a reduction in intracellular reactive oxygen species (ROS) [188]. More specifically, resveratrol enhances both the expression and activity of key antioxidant enzymes, including superoxide dismutase (SOD), catalase, and glutathione peroxidase, resulting in mitochondrial accumulation of hydrogen peroxide and the induction of apoptosis in tumor cells. Additionally, resveratrol inhibits the accumulation of hypoxia-inducible factor-1α (HIF-1α), suppressing glycolytic metabolism and interfering with the metabolic reprogramming that sustains tumor growth. On the contrary, in normal cells, a protective role of resveratrol has been reported by mildly upregulating antioxidant defense systems and reducing oxidative stress, thereby preserving cellular integrity [188].

It also modulates different signaling pathways involved in cell proliferation, apoptosis, angiogenesis, and metastasis. For instance, resveratrol activates the tumor suppressor p53, inhibits NF-κB and STAT3 signaling, and interferes with the PI3K/AKT and MAPK pathways. These molecular effects have been observed in many cancer cell lines, such as breast, prostate, colon, lung, and glioblastoma [189,190]. In breast cancer cells, resveratrol was shown to inhibit estrogen receptor α (ERα) gene expression via phosphorylation of the NF-Y/p53/Sin3/HDAC1 complex, mediated by p38MAPK/CK2 signaling, including MCF-7 tamoxifen-resistant cells. IC_50_ values for viability were in the 30–45 μM range [191]. In glioblastoma, the combination of resveratrol with Notch inhibitors induced cell death by shifting the balance from autophagy to apoptosis. Resveratrol activated ERK1/2 at low concentrations (1 pM-10 μM), but at higher concentrations (50–100 μM), it inhibited MAPK in human neuroblastoma SH-SY5Y cells [192]

In vivo studies corroborate the in vitro findings, showing that resveratrol can inhibit tumor growth, reduce metastatic spread, and improve survival in animal models of cancer (0.5, 5, or 50 mg/kg BW; oral gavage) [193]. In mouse models, it has demonstrated the ability to reduce tumor volume in breast and colon cancer and to limit metastasis in prostate and lung cancers. These effects are associated with its regulation of angiogenesis, modulation of the tumor microenvironment, and enhancement of immune response [194].

Despite its promising preclinical profile, translating resveratrol into clinical therapy remains challenging. Although topical application of the compound has proven effective in inhibiting tumor formation in animal models of skin cancer, its therapeutic use against other tumor types is limited by the need for oral administration. Indeed, clinical trials have confirmed that resveratrol’s therapeutic potential is hampered by poor pharmacokinetic profile and low bioavailability after oral consumption. Resveratrol undergoes rapid metabolism in the intestine and liver, resulting in low plasma concentrations and limited systemic exposure [195].

To overcome this limitation, numerous efforts have been made to identify strategies capable of enhancing the bioavailability of resveratrol following oral administration. Among these, the use of delivery systems such as nanoformulations, liposomes, solid lipid nanoparticles, and micellar systems has shown particular promise, as they can improve the compound’s solubility, stability, and absorption, thereby increasing its systemic availability and therapeutic potential [187]. One promising formulation is JOTROL™, a micellar 10% resveratrol solubilization formulation that is thought to increase bioavailability of resveratrol via lymphatic system absorption by exploiting lymphatic distribution to bypass first-pass metabolism and enhance systemic bioavailability. Early clinical data indicate that JOTROL™ achieves significantly higher plasma levels of resveratrol compared to conventional formulations [195]. Additionally, co-administration with bioenhancers, use of prodrugs, and structural analog development are under investigation as means to optimize its therapeutic use [196,197].

Moreover, several studies have demonstrated that resveratrol can enhance the efficacy of conventional chemotherapeutic agents such as 5-fluorouracil, doxorubicin, temozolomide and cisplatin by promoting apoptosis and inhibiting tumor cell proliferation. At the same time, resveratrol exerts protective effects against drug-induced toxicity in healthy tissues, particularly in the heart, highlighting its potential as an adjuvant in cancer therapy (5-FU alone (200 μg/mL), resveratrol alone (200 μg/mL), and 5-FU in combination with resveratrol (200 μg/L + 50 μmol/L)) [198,199,200,201].

In conclusion, resveratrol represents a compelling candidate for cancer chemoprevention and adjunctive therapy, owing to its pleiotropic actions on cancer-related pathways. However, to fully realize its clinical potential, further work is needed to address the challenges of bioavailability and to validate its efficacy in well-designed, large-scale human trials [202].

## 4. Conclusions

**Figure 1:**
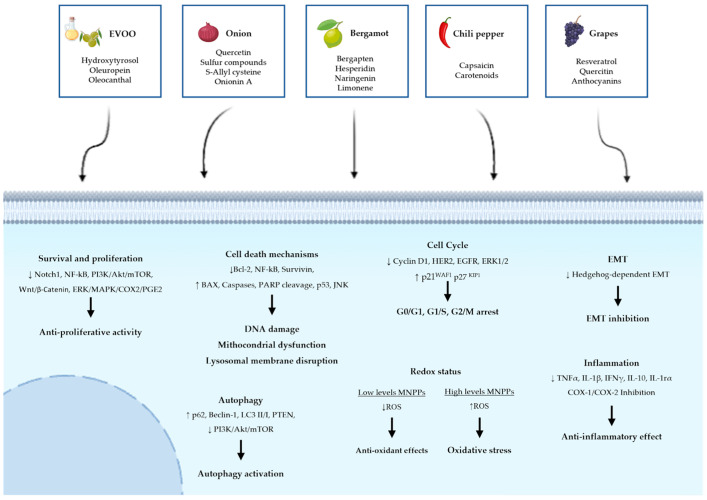
Common Molecular Mechanisms underlying the antitumor effects of five Selected Mediterranean Natural Plant-Derived Compounds. The figure summarizes the effects of selected Mediterranean plant extracts on cancer-related cellular pathways. Arrows indicate the upregulation (↑) or downregulation (↓) of key molecular markers and pathways.

Natural compounds are generally less toxic or even non-toxic to normal cells while exhibiting selective activity against cancer cells. They present a favorable safety profile that, coupled with the richness of phytochemical diversity, allows them to modulate multiple oncogenic signaling pathways simultaneously. Increasing evidence supports their role not only in cancer prevention but also as adjuvant to conventional chemotherapy, enhancing therapeutic efficacy, reducing drug resistance, and mitigating adverse side effects. Many phytochemicals act by interfering with key molecular hallmarks of carcinogenesis, such as sustaining proliferative signaling (e.g., via EGFR or PI3K/AKT/mTOR pathways), evading apoptosis (e.g., through BAX/Bcl-2 ratio modulation), inducing autophagy or ferroptosis, inhibiting angiogenesis, and preventing metastasis (e.g., through EMT suppression). Others restore immune surveillance, regulate oxidative stress, and influence the tumor microenvironment. These mechanisms make natural compounds particularly promising as multi-targeted therapeutic agents, complementing standard anticancer treatments. Notably, many of these compounds, such as oleocanthal, oleuropein, quercetin, onionin A, capsaicin, bergapten, and resveratrol, exhibit potent antioxidant activity, reducing oxidative stress, thus contributing to cancer prevention by maintaining genomic stability and limiting inflammation-induced tumor initiation. However, this same antioxidant action must be evaluated carefully in the therapeutic context, as it may interfere with ROS-dependent cancer treatments (Figure 1). This paradox is called the “antioxidant double-edged sword” [203]. A major advantage of these natural compounds lies in their abundance in common plants; they can be easily extracted from readily available plants, which has a considerable effect on reducing the cost of production. Despite their promising potential in cancer therapy and prevention, several challenges remain. These include variability in phytochemical composition, the need for rigorous standardization and clinical validation, and the risk of herb–drug interactions. Therefore, further preclinical and translational studies are essential to elucidate their mechanisms of action and improve bioavailability. Due to low solubility, poor absorption, or rapid metabolism, their therapeutic effectiveness is currently limited. In addition, it is important to evaluate safety profiles in well-designed clinical trials.

In conclusion, it is evident that a diet abundant in vegetables, fruit, olive oil, and herbs, like the Mediterranean diet, associated with physical activity and a reduced intake of sugar and alcohol, may play a role not only in preventing but also in supporting the treatment of malignancies. The integration of nutritional strategies and phytochemicals with conventional therapeutic modalities may offer a more comprehensive and efficacious approach to contemporary oncology.

## Figures and Tables

**Table 1:** Anticancer Properties of Key Bioactive Compounds Found in Major Mediterranean Plants.

Food Source	Key Bioactive Compounds	Antitumoral Mechanisms	Cancer Types Studied	References
Olive (EVOO)	Hydroxytyrosol, oleuropein, oleocanthal	ROS scavenging, apoptosis induction, anti-inflammatory, inhibition of MMPs and COX-2	Breast, colon, prostate	[45,46]
Grapes (Red/Black)	Resveratrol, quercetin, anthocyanins	Inhibits angiogenesis, induces apoptosis, suppresses estrogen receptors, modulates p53	Breast, colon, lung	[47,48]
Citrus fruits	Hesperidin, naringenin, limonene, bergapten	Detoxification enzyme activation, anti-proliferative, apoptosis, antioxidant	Colon, gastric, liver, breast	[49,50]
Onion	Quercetin, sulfur compounds (allicin-like)	DNA protection, anti-inflammatory, apoptosis induction, HDAC inhibition	Stomach, colon, prostate	[51]
Pepper (Capsicum)	Capsaicin, carotenoids (lutein, β-carotene)	Apoptosis via mitochondrial pathway, anti-inflammatory, antioxidant	Prostate, gastric, pancreatic	[52]
Rosemary	Carnosic acid, rosmarinic acid, ursolic acid	Inhibits tumor promotion, ROS scavenging, cell cycle arrest, modulates MAPK and PI3K/Akt	Breast, skin, liver	[53]
Oregano	Carvacrol, thymol, rosmarinic acid	Cytotoxicity in cancer cells, NF-κB inhibition, apoptosis induction, antioxidant	Colon, breast, lung	[54,55]
Basil	Eugenol, ursolic acid, apigenin	Inhibits angiogenesis, induces apoptosis, modulates NF-κB and PI3K/AKT signaling	Breast, colon, lung	[56]

**Table 2:** Comparison of Five Selected Mediterranean Plant Species with Their Non-Mediterranean Counterparts.

Plant/Product	Region	Representative Compounds	Concentration Range/Quantitative Data	DPPH IC_50_ (µg/mL or µg/mL Equivalent)	Analytical Method (Typical)	References
Olive (EVOO)	Mediterranean (*Olea europaea)*	Oleuropein, Hydroxytyrosol, Squalene	Oleuropein: 0.5–2% DW; Hydroxytyrosol: 50–200 mg/kg oil; Squalene: up to 7%	DPPH IC_50_ = 25–40 µg/mL	HPLC-DAD; LC-MS/MS	[57,58]
	Non-Mediterranean (*Elaeis guineensis*)	β-carotene, Tocotrienols	β-carotene: 500–700 mg/kg; Tocotrienols: 200–300 mg/kg	DPPH IC_50_ = 70–100 µg/mL	HPLC; UV-Vis	[59]
Grapes (Red/Black)	Mediterranean (*Vitis vinifera*)	Resveratrol, Quercetin, Malvidin-3-glucoside	Resveratrol: 1–7 mg/L wine; Quercetin: 2–10 mg/L	DPPH IC_50_ = 15–35 µg/mL	HPLC; Folin–Ciocalteu	[60,61]
	Non-Mediterranean (*Vitis labrusca*)	Delphinidin derivatives	Delphinidin derivatives: 5.13–80.89 mg/L	DPPH IC_50_ ≈ 2.22 mg/L	HPLC	[62]
Citrus fruits	Mediterranean (*C. limon*, *C. sinensis*, *C. aurantium*)	Hesperidin, Limonene, Ascorbic acid	Hesperidin: 20–60 mg/100 g FW; Limonene: 70–90% EO; Vitamin C: 40–60 mg/100 g	DPPH IC_50_ = 20–40 µg/mL	HPLC; GC-MS; UV-Vis	[63]
	Non-Mediterranean (*C. reticulata*, tropical Asia)	Naringin, γ-Terpinene	Naringin: 10–40 mg/100 g; γ-terpinene: 10–20% EO	DPPH IC_50_ = 50–70 µg/mL	HPLC; GC-MS	[64]
Onion	Mediterranean (*Allium cepa*)	Quercetin, Allicin derivatives	Quercetin: 100–300 mg/kg FW; Allicin: up to 0.5 mg/g	DPPH IC_50_ = 40–55 µg/mL	HPLC-DAD; spectrophotometry	[51,65]
	Non-Mediterranean (*Allium fistulosum*)	Allyl sulfides	Total phenolics < 80 mg GAE/100 g	DPPH IC_50_ ≈ 70 µg/mL	HPLC; UV-Vis	[66]
Pepper (Capsicum)	Mediterranean (*C. annuum*)	Capsaicin, Lutein, Zeaxanthin	Capsaicin: 0.1–1.0%; Carotenoids: 50–200 µg/g FW	DPPH IC_50_ = 30–50 µg/mL	HPLC-DAD; spectrophotometry	[67,68]
	Non-Mediterranean (*C. annum and C. chinense*)	Dihydrocapsaicin, Capsaicin	Dihydrocapsaicin, Capsaicin: 0.1 to 10 μg/mL	DPPH IC_50_ = 18.04 µg/mL	HPLC	[69]

## Data Availability

No new data were created or analyzed in this study.

## Abbreviations

The following abbreviations are used in this manuscript:

MNPPs	Mediterranean Natural Plant Products
EGCG	EpiGalloCatechin Gallate
MDR	MultiDrug Resistance
MD	Mediterranean Diet
EVOO	Extra Virgin Olive Oil
EPIC	European Prospective Investigation into Cancer and Nutrition
MMPs	Matrix MetalloProteinases
COX2	CycloOXygenase-2
HGF	Hepatocyte Growth Factor
EGFR	Epidermal Growth Factor Receptor
IGF-1R	Insulin-like Growth Factor 1 Receptor
PARP	Poly (ADP-ribose) Polymerase
MUFA	Monounsaturated Fatty Acid
HER2	Human Epidermal Growth Factor Receptor 2
CDK1	Cyclin Dependent Kinase 1
CDK2	Cyclin Dependent Kinase 2
SOD	Superoxide Dismutase
JNK	Jun-N-Kinase
ROS	Reactive Oxygen Species
NF-κB	Nuclear Factor kappa-light-chain-enhancer of activated B cells
PI3K	PhosphatidylInositol 3-Kinase
ERK	Extracellular Signal-Regulated Kinase
STAT3	Signal Transducer and Activator of Transcription 3
MDSCs	Myeloid-Derived Suppressor Cells
PTEN	Phosphatase and TENsin homolog
ER	Estrogen Receptor
NSCLC	Non-Small Cell Lung Cancer
TRPV1	Transient Receptor Potential Cation Channel Subfamily V member 1
